# Computational methods for alignment and integration of spatially resolved transcriptomics data

**DOI:** 10.1016/j.csbj.2024.03.002

**Published:** 2024-03-05

**Authors:** Yuyao Liu, Can Yang

**Affiliations:** a Department of Automation, School of Information Science and Technology, Tsinghua University, Beijing, China; b Department of Mathematics, The Hong Kong University of Science and Technology, Hong Kong, China

**Keywords:** Spatially resolved transcriptomics, Slices alignment, Data integration, Batch effects

## Abstract

Most of the complex biological regulatory activities occur in three dimensions (3D). To better analyze biological processes, it is essential not only to decipher the molecular information of numerous cells but also to understand how their spatial contexts influence their behavior. With the development of spatially resolved transcriptomics (SRT) technologies, SRT datasets are being generated to simultaneously characterize gene expression and spatial arrangement information within tissues, organs or organisms. To fully leverage spatial information, the focus extends beyond individual two-dimensional (2D) slices. Two tasks known as slices alignment and data integration have been introduced to establish correlations between multiple slices, enhancing the effectiveness of downstream tasks. Currently, numerous related methods have been developed. In this review, we first elucidate the details and principles behind several representative methods. Then we report the testing results of these methods on various SRT datasets, and assess their performance in representative downstream tasks. Insights into the strengths and weaknesses of each method and the reasons behind their performance are discussed. Finally, we provide an outlook on future developments. The codes and details of experiments are now publicly available at https://github.com/YangLabHKUST/SRT_alignment_and_integration.

## 1 Introduction

Most of the complex biological regulatory activities happen in three dimensions (3D). To comprehensively master the principles and theories behind these activities, it requires not only deciphering the molecular profiles of cells but also comprehending the influence of their spatial context [1]. Technologies for spatial omics [1], [2], [3], [4], [5], [6], [7], [8], [9], [10], [11], [12], [13], [14], [15], [16], [17], [18], [19] measure molecular parameters of tissue samples and provide corresponding spatial information, enabling analyses utilizing both cell identity and the surrounding microenvironment. Serving as a crucial branch in spatial omics, spatially resolved transcriptomics (SRT) enables the establishment of a connection between tissue biology and genomics [20]. It allows a range of analyses for diverse biological systems by concurrently leveraging mRNA expression profiles and spatial coordinates [21], [22], [23]. SRT technologies can be mainly classified into two types: image-based SRT technologies and sequencing-based SRT technologies [24]. The former, which includes seqFISH [5], osmFISH [6], MERFISH [7], etc., has evolved from single-molecule florescence in situ hybridization (smFISH) [25], [26], [27] and can measure tens to thousands of RNAs at the single-cell level. It provides a cell-by-gene matrix for each measured tissue slice, coupled with spatial coordinates for cells, as the output SRT data, where the gene expression patterns of cells are represented as row vectors. The latter, represented by Visium [8], Slide-seqV2 [11] and Stereo-seq [12], possess the ability for unbiased sequencing of RNA species at the whole transcriptome level but are limited by the low capturing rate of mRNAs. In contrast, it generates a spot-by-gene matrix and spatial coordinates for spots for each slice. Each spot may contain multiple cells, with the quantity of cells contained depending on the resolution of the specific technology. Hence, the measured gene expression profiles for each spot correspond to the aggregation of cells with that spot.

Merely analyzing and extracting information from a 2D perspective, or overlooking the importance of spatial information, will lead to the underutilization of the true value inherent in SRT data, significantly restricting the interpretation of biological processes [1]. Therefore, two typical tasks, slices alignment and data integration, are introduced for 3D SRT data analysis in order to enhance the effectiveness of downstream analyses and provide a more comprehensive insight into measured tissue samples while fully leveraging the spatial information of tissue slices. Slices alignment involves transforming different tissue slices into a common coordinate system (CCS). Due to measurement errors, spatial warping between different slices is inevitable [28]. As a result, measured spatial coordinates exhibit variations among slices, making them unsuitable for direct joint analysis. Transforming different slices into the same CCS enables meaningful comparisons across slices or across biological scales [29]. Aligning adjacent slices from the same tissue to a CCS further allows for comprehensive 3D structure reconstruction which facilitates the interpretation of the relationship between gene expression and function at the whole tissue level [30], [31]. Data integration is the problem of combining information from different sources to provide a unified perspective, yielding more comprehensive, systems-level insights in biology [32], [33], [34]. It typically involves mapping slices from individual datasets or multiple datasets to the same feature space [35], where similar types of cells or spots cluster together in close proximity, while distinct types are positioned farther apart. To achieve this, addressing batch effects caused by variations in samples, technologies, measurement environments, or operational errors is crucial. Numerous computational methods for the integration of SRT data have been developed [35], [36], [37], [38], [39], [40], [41], [42], [43]. These methods often employ method-specific approaches to create a feature space that preserves biological variation and mitigates batch effects, enhancing the effectiveness of joint clustering and spatial domain identification tasks using SRT data. It is worth mentioning that slices alignment and data integration of SRT data are usually performed on datasets with relatively higher number of measured genes and lower spatial resolutions, which are generated by sequencing-based SRT technologies [8], [9], [10], [11], [12].

To date, there has been a lack of a systematic comparison and analysis of the underlying reasons for the effectiveness of alignment and integration methods for SRT data. In this review, we aim to address this gap by examining representative methods for both slices alignment [28], [44], [45] and data integration [41], [43], [44] tasks while comparing their performance. We begin by delving into the details and principles of these methods, providing a comprehensive understanding of their inner workings. Subsequently, we evaluate the performance of these methods by applying them to various SRT datasets for testing, specifically focusing on their efficacy in representative downstream tasks. Through this analysis, we offer valuable insights into the strengths and weaknesses of each method, shedding light on the factors contributing to their performance disparities. Finally, we discuss potential future directions and opportunities for improvements in this field.

## 2 Representative methods for alignment and integration of SRT data

Numerous methods have been introduced to address slices alignment and data integration tasks using SRT data, aiming for a more comprehensive understanding about the principles behind biological processes, cell interactions within tissues and mechanisms of diseases. In this section, we highlight five representative methods [28], [41], [43], [44], [45] which we have tested on several SRT datasets. The results will be displayed and analyzed in the subsequent sections.

PASTE [44] provides two methods to tackle both alignment and integration tasks which we refer to as PASTE_alignment and PASTE_integration for clarity. PASTE_alignment is a method that computes pairwise alignments of slices using fused Gromov-Wasserstein optimal transport [46]. It finds an optimal probabilistic mapping (Π) between spots in each slice of a slice-pair to be aligned. It takes into account both transcriptional dissimilarity represented by the variation in gene expression between aligned spots from different slices, and difference in spatial distances represented by the physical distance of spatial coordinates between pairs of aligned spot from the same slice. After aligning a pair of consecutive tissue slices, PASTE_alignment solves a weighted Procrustes problem [47], [48], defined by using spatial coordinates of the original slices and the optimal probabilistic mapping, to find a rotation and a translation for one of the slices. By repeating this procedure for each pair of adjacent slices, the spatial coordinates of all slices can be transformed into a same CCS, enabling the reconstruction of the 3D structure of the tissue. Given a set of SRT slices, PASTE_integration calculates the gene expression matrix for a single center slice, using one of the slices as the template, to represent the integration of multiple slices. Operating under the biological assumption that spots often correspond to a limited number of cell types or cell states, the center slice is reasonably assumed to have a low-rank transcript count matrix. This is achieved by solving a problem that combines a fused Gromov-Wasserstein barycenter [46] with non-negative matrix factorization (NMF) [49].

PASTE2 [45] is an extension of the PASTE algorithm designed specifically for aligning partially overlapped SRT slices. Unlike its predecessor, PASTE2 focuses solely on the alignment task and does not incorporate a method for data integration. It introduces a novel formulation called the partial fused Gromov-Wasserstein optimal transport problem, which takes into account a parameter "*s*" representing the overlap percentage between a pair of slices to be aligned. The key idea behind PASTE2 is to allow for a dynamically changing alignment ratio between different pairs of slices based on the determined overlap percentage. To select the appropriate value of "*s*", a model selection procedure is employed, where various values of "*s*" are tested by running PASTE2. The final selection of "*s*" which represents the actual overlap percentage, is determined using an edge inconsistency score. [45] This score measures the spatial coherence of a graph that consists of two clusters of nodes: one cluster containing spots that are aligned and another cluster containing spots that are not aligned.

GPSA [28] is a deep Gaussian process (DGP) method [50] specifically designed to align SRT slices onto a CCS. It comprises two Gaussian process (GP) [51] layers that work together to achieve alignment. The first layer represents the warping functions for different slices, mapping the spatial coordinates of each spot to their corresponding location in the CCS. The second layer models gene expression readouts, leveraging the new coordinates of spots given by the first layer. By integrating the gene expression data with the aligned coordinates, GPSA enables the reconstruction of the 3D structure of the tissue, providing valuable insights into the relationship between gene expression and spatial organization. It is worth noting that GPSA is not limited to 2D coordinates typically found in SRT data; it also has the potential to handle 3D coordinate systems or even 4D spatiotemporal coordinate systems [28]. This flexibility makes GPSA a versatile tool for aligning SRT slices across different spatial dimensions, allowing for comprehensive analysis and interpretation of complex biological structures.

STAligner [41] is primarily an integration method for SRT data, but it also includes an alignment procedure that enables the reconstruction of the 3D tissue structure. The key component of STAligner is a graph attention autoencoder [52], which serves as the underlying model structure. This model combines both transcriptional information and spatial location information to achieve effective integration. The encoder in STAligner maps the preprocessed gene expression matrices of SRT slices to a shared latent space with lower dimensionality. By leveraging the spatial coordinates of spots, STAligner creates a spatial neighbor graph for each 2D slice. This graph captures the spatial relationships between spots, which are then used to learn spatially aware embeddings in the latent space through the model. Additionally, STAligner constructs spot triplets that consist of anchor-positive and anchor-negative spot pairs. These pairs are defined based on the representations of spots in the latent space. The method introduces spot triplet learning, which involves minimizing the distance between the anchor-positive pair and maximizing the distance between the anchor-negative pair. This approach helps to mitigate batch effects in the latent space, enhancing the integration process [41].

STitch3D [43] is a recently developed data integration method that simultaneously addresses spatial domain identification and cell-type deconvolution tasks. The method begins with a preprocessing procedure that involves aligning the SRT slices using either iterative closest point (ICP) [53] or PASTE [44]. This alignment step maps the coordinates of spots onto a CCS, enabling the construction of a 3D neighbor graph for the entire tissue based on the established CCS. In STitch3D, the 3D graph, gene expression matrices of SRT slices, and a cell-type-specific gene expression profile are used as inputs for the model. To account for batch effects and capture the biological variation in the latent space, STitch3D introduces two sets of parameters: slice- and spot-specific effects, as well as slice- and gene-specific effects. These parameters, along with a slice-specific decoder, facilitate the reconstruction of gene expressions for each spot. By considering these parameters and employing the slice-specific decoder, STitch3D effectively mitigates batch effects and distills the biological variation in the latent space [43]. The outputs of STitch3D can be used for various downstream tasks, including spatial trajectory inference, denoising of low-quality gene-expression measurements, and generation of virtual tissue slices.

## 3 Experimental results

### 3.1 Results with slices alignment methods

We evaluated four methods for the task of slice alignment: PASTE_alignment [44], PASTE2 [45], GPSA [28], and STAligner [41]. While the first three methods were originally developed specifically for slice alignment, STAligner is primarily designed for integrating data across multiple slices. However, STAligner also offers a landmark-based approach to slice alignment, which motivated us to include it in our evaluation.

The slice alignment method in STAligner involves selecting landmark regions between two slices based on prior knowledge. These regions serve as the reference for alignment. Subsequently, within the chosen landmark regions, spot pairs are identified using the mutual nearest neighbors (MNN) algorithm in the latent space, where batch effects have been eliminated. Each pair of spots, belonging to different slices, is then utilized to align the slices and achieve a transformation of spatial coordinates using the ICP algorithm. It is important for the landmark domains of the two slices to have sufficient overlap and typically have asymmetrical shapes. This is because the ICP algorithm may misalign two domains with symmetrical shapes by incorrectly rotating one of them, particularly in datasets with noise. To mitigate the potential impact of different selections of landmark domains on the alignment results, we decided to use the entire slice as the landmark. MNN spot pairs were identified using the low-dimensional representation of all spots, and alignment was performed using the ICP algorithm.

It is worth mentioning that slight differences exist between methods during the data preprocessing stage, as they are adjusted to better suit their respective models. However, to maintain the original operating procedure of each method as much as possible, we standardized the spot filtering process across all methods. This ensured that, for the same dataset, each algorithm processed the same number and identity of spots. All other preprocessing steps and hyperparameters were kept consistent with the original methods to maintain comparability.

**Fig. 1:**
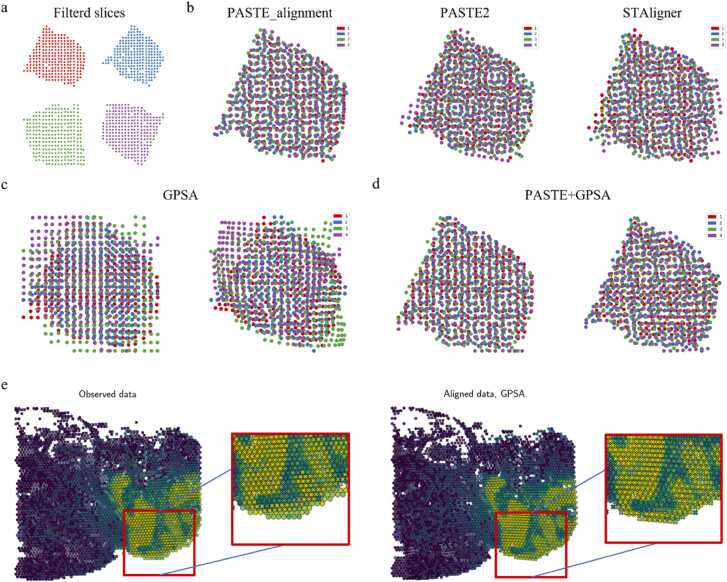
Experimental results with slices alignment methods tested on a breast cancer dataset [8] and a mouse brain dataset [54], [55] for qualitative analysis. a, The display of SRT slices of the breast cancer dataset after filtering spots and genes. The dataset comprises four slices, with spots in different slices represented by different colors. The dataset has a relatively small scale, with each slice containing 250 to 263 spots and 7453 to 7998 genes. A high percentage of coverage and a noticeable rotation can be found between different slices. **b,** The results of aligning four slices of the breast cancer dataset using PASTE_alignment, PASTE2, and STAligner. PASTE_alignment shows the best alignment performance overall. PASTE2 and STAligner both exhibits some alignment deviations for the third and fourth slices, while STAligner also demonstrates alignment discrepancies between the first and second slices. **c,** Visualization of the four slices being directly overlaid after scaling their original coordinates to the same range (left) and the alignment of them with GPSA(right). GPSA exhibits poor alignment capability in this condition. **d,** Pre-alignment of slices with PASTE_alignment (left) after leveraging the data preprocessing procedure provided by GPSA, followed by further alignment using GPSA (right). GPSA fine-tuned the alignment based on the pre-alignment by PASTE. **e,** The display of the two serial sections of the mouse brain dataset (left), where the spots are colored due to the expression value of Pcp2. Spots from the two different slices are represented by ‘x′ and ‘o′, respectively. GPSA was applied to align the two slices (right).

We first evaluated the slice alignment performance of these methods using a breast cancer dataset sourced from Ståhl et al. [8]. This dataset consists of four SRT slices and has relatively small numbers of spots and genes. Each slice contains 250 to 263 spots and 7453 to 7998 genes. Notably, there is a high degree of coverage and noticeable rotation between the different slices (Fig. 1a). Among the evaluated methods, PASTE_alignment demonstrated the best performance, accurately aligning the different slices. PASTE2 successfully aligned the first three consecutive slices, achieving results similar to PASTE_alignment. However, it exhibited some deviations when aligning the third and fourth slices. This discrepancy arises from PASTE2's requirement to estimate the overlap percentage "*s*" between two slices before alignment, which influences the alignment outcome. In this dataset, the estimated "*s*" by PASTE2 significantly deviates from the true overlap percentage. PASTE2 selects the value of "*s*" during the model selection procedure based on their defined edge inconsistency score. However, the criteria for selecting "*s*" is determined empirically and lack sufficient theoretical basis. Consequently, significant discrepancies between the estimated "*s*" and the true overlap percentage can arise for certain datasets. In the case of this dataset, the estimated "*s*" for each pair of adjacent slices is not greater than 0.5, with the estimation between the third and fourth slices being particularly small, which is clearly unreasonable and results in suboptimal alignment. Manually setting the overlap percentage "*s*" between slices to 0.99 yields results similar to PASTE_alignment. The alignment results of STAligner exhibit some deviations between the first and second slices, as well as the third and fourth slices. This discrepancy arises because STAligner relies on MNN pairs defined in the latent space for slice alignment. However, STAligner fails to find a sufficient number of MNN pairs. For each pair of slices, fewer than 20% of spots find a corresponding MNN spot, leading to inaccurate coordinate transformations (Fig. 1b).

Slice Alignment based on the original spot coordinates using GPSA (Fig. 1c) exhibits distinct differences compared to the previous three methods. In fact, GPSA fails to align these four slices. This discrepancy arises from the underlying assumption of GPSA, which assumes that substantial spatial warping does not occur between different slices. As a result, GPSA expects spots to be aligned between different slices to have similar coordinates. To enforce this assumption, the mean function of the GP prior for each spot in the first GP layer is set to its true coordinates. This configuration aims to prevent extreme warping during coordinate transformations and preserve the original spatial relationships within the data. Consequently, after transforming different slices to a CCS, each spot is mapped to a new position centered around its true coordinates. However, in the case of this breast cancer dataset, clear rotation exists between slices, and GPSA lacks the capability to align slices with significant warping. To overcome this limitation, we utilized PASTE_alignment to pre-align these four slices after applying the data preprocessing procedure provided by GPSA. Subsequently, we further aligned the pre-aligned slices using GPSA (Fig. 1d). We observed that GPSA fine-tuned the pre-alignment obtained from PASTE_alignment, resulting in improved alignment accuracy and accounting for the rotation present in the dataset.

Subsequently, we evaluated these algorithms using a mouse brain dataset [54], [55] consisting of two adjacent slices. Due to the substantial overlap between these two slices in the original dataset, all four methods successfully aligned the two slices, with minimal variation in alignment performance among different methods. However, it is important to note that, as GPSA is based on GPs, only GPSA achieves more flexible non-rigid transformations of coordinates (Fig. 1e). In contrast, the other methods can only achieve rigid transformations through translation and rotation.

**Fig. 2:**
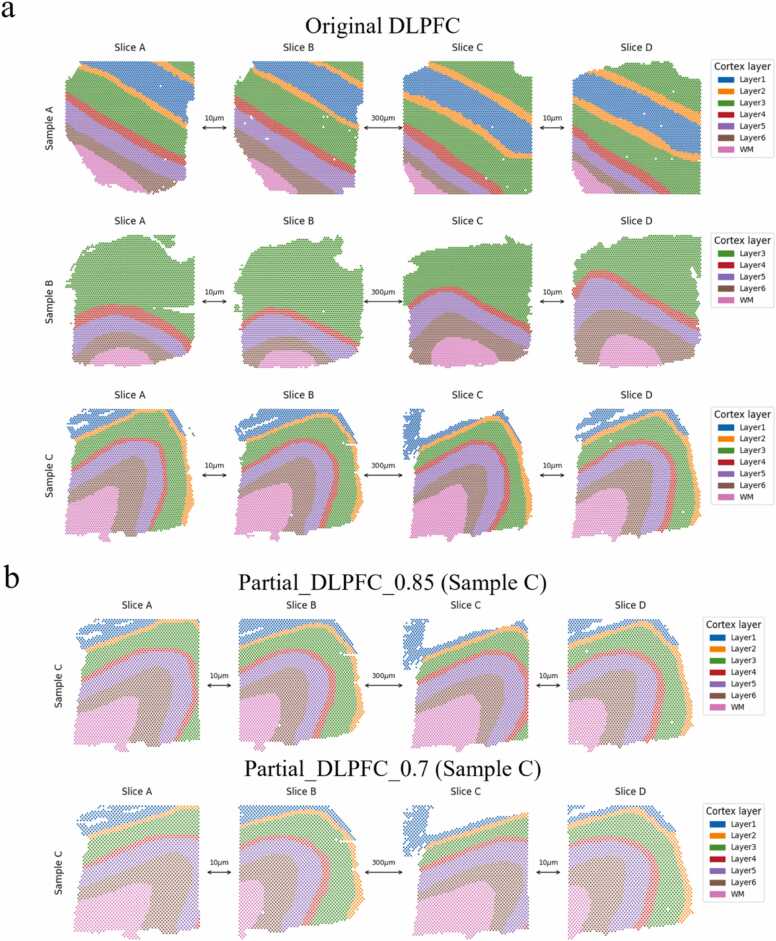
Display of the DLPFC dataset [56] and the partially overlapped datasets generated based on it. a, Display of the DLPFC dataset which comprises three samples from three human individuals, with each sample consisting of four slices. The distance between slices A and B, as well as between slices C and D, is 10 µm. However, the distance between slice B and slice C is 300 µm. The spots are colored based on manual annotation which classifies spots into six neocortical layers and white matter (WM). Samples A and C contain spots from all seven categories, while Sample B only includes five of these categories. **b, c,** The display of sample C in partial_DLPFC_0.85 (top) and partial_DLPFC_0.7 (bottom) which are partially overlapped datasets generated from DLPFC dataset. For the partial_DLPFC_0.85 dataset, based on the numerical values of horizontal coordinates, 85% of the spots on the left half of slice A and C as well as 85% of spots on the right half of slice B and D are retained. The procedure is similar for partial_DLPFC_0.7, where the retained proportion of spots is changed to 70%.

In order to conduct a quantitative analysis of these slice alignment methods, we further employed a human dorsolateral prefrontal cortex (DLPFC) [56] dataset (Fig. 2a). This dataset comprises three samples from three human individuals, with each sample consisting of four slices. The distance between slices A and B, as well as between slices C and D, is 10 µm. However, the distance between slice B and slice C is 300 µm. In this dataset, spots have been manually annotated into seven categories, including six neocortical layers and white matter (WM). Samples A and C contain spots from all seven categories, while Sample B only includes five of these categories. Additionally, to test the alignment performance of different methods on partially overlapped slices, we manually created two datasets, namely partial_DLPFC_0.85 and partial_DLPFC_0.7 (Fig. 2b), based on DLPFC. For the partial_DLPFC_0.85 dataset, 85% of the spots on the left half of slices A and C, as well as 85% of spots on the right half of slices B and D, are retained based on the numerical values of horizontal coordinates. This implies that two originally identical adjacent slices will have a 70% overlap after cropping. The procedure is similar for partial_DLPFC_0.7, where the retained proportion of spots is changed to 70%.

**Fig. 3:**
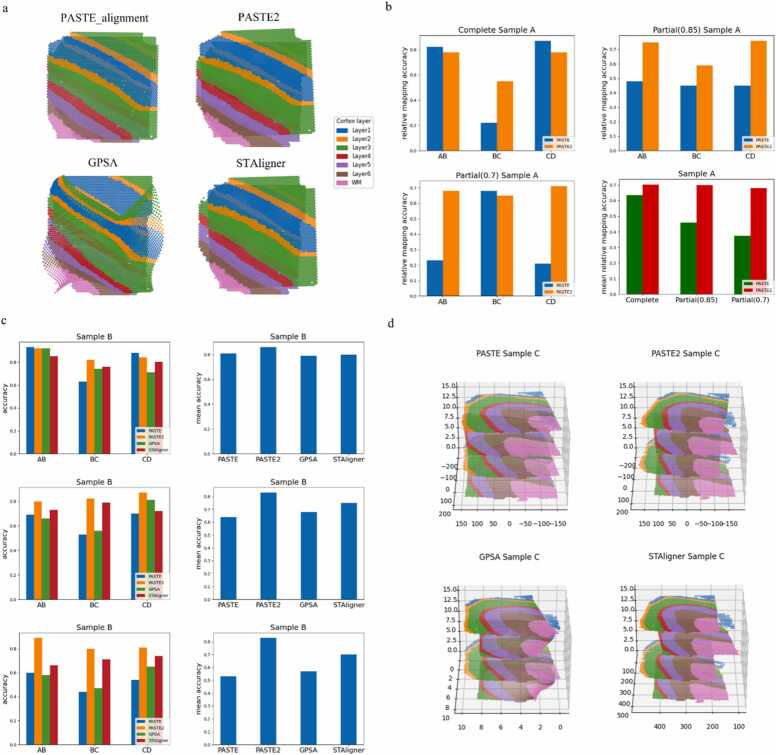
Experimental results with slices alignment methods tested on DLPFC dataset [56] for quantitative analysis. a, Alignment of sample A from DLPFC dataset using PASTE_alignment, PASTE, GPSA and STAligner. The four slices of sample A are stacked together. PASTE2 and STAligner show better alignment of slice B and C, while GPSA and PASTE exhibit misalignment of these two slices, which are further apart in the horizontal scale. **b,** Using the relative mapping accuracy score derived from the mapping accuracy introduced by PASTE to compare the alignment results of PASTE_alignment and PASTE2. The accuracies between each pair of consecutive slices of sample A from DLPFC (top-left), partial_DLPFC_0.85 (top-right) and partial_DLPFC_0.7 (bottom-left) and the mean relative mapping accuracies (bottom-right) are shown. **c,** The comparison of the alignment results of all four alignment methods using the coordinate-based accuracy score. The accuracies between each pair of consecutive slices and the mean accuracies of sample B from DLPFC (top), partial_DLPFC_0.85 (middle) and partial_DLPFC_0.7 (bottom) are displayed. **d,** The 3D visualization of the alignment results of sample C from the partial_DLPFC_0.85 dataset.

We aligned each of the three samples separately. From the alignment results of sample A (Fig. 3a), we observed that PASTE_alignment demonstrated good performance in aligning slice A with slice B and slice C with slice D. However, it showed suboptimal alignment between slice B and slice C. Notably, there was a significant distance between slice B and slice C in each sample, resulting in a noticeable offset between them in two out of three samples, except for sample C (Fig. 2b). Unfortunately, PASTE_alignment did not effectively correct this offset. On the other hand, both PASTE2 and STAligner partially addressed this offset issue. PASTE2 achieved this by estimating an overlap percentage smaller than 1, which prevented excessive overlap between spots from different slices. Similarly, STAligner limited the influence of improper spots on the coordinate transformation process by selecting a relatively low proportion of MNN spot pairs between different slices. Since these MNN pairs are defined in the latent space, this method effectively reduced the interference of batch effects, resulting in more accurate spot pairing. When aligning slices using GPSA, we used slice B as the template. Consequently, while the shape of slice B remained unchanged, slice C underwent substantial deformation to map to the common coordinate system represented by slice B. Similarly, slice D, which initially had high overlap with slice C, also transformed into a shape similar to slice C. However, this alignment approach did not yield satisfactory results. Utilizing the mapping accuracy score provided by PASTE, we introduced a relative mapping accuracy score to quantitatively compare the alignment effects of PASTE_alignment and PASTE2 on datasets with varying overlap percentages (Fig. 3b). The relative mapping accuracy score utilizes the optimal probabilistic mapping matrix generated by PASTE_alignment and PASTE2 after aligning adjacent slices. It calculates the sum of matching weights for spot types belonging to the same category in both slices, divided by the estimated overlap percentage "*s*" for the two slices, serving as the relative mapping accuracy score. Notably, the sum of the parameters of the optimal probabilistic mapping matrix output by PASTE2 is set to "*s*". Therefore, the estimated overlap percentage for PASTE_alignment was set to 1.

Analyzing the results for sample A, we found that the accuracy of PASTE_alignment in aligning slices A with B and slices C with D in the DLPFC dataset was slightly higher than that of PASTE2. However, PASTE2 demonstrated significantly better performance in aligning slices B with C. In partially overlapped datasets, PASTE2 generally exhibited higher accuracy in almost all cases, except for the alignment results of slices B with C in the partial_DLPFC_0.7 dataset, where PASTE_alignment surpassed PASTE2. This outcome can be attributed to randomness, as the originally low overlap percentage between the two slices increased after cropping. Furthermore, we calculated the mean relative mapping accuracy for each sample by averaging the accuracy scores for each pair of consecutive slices. It was observed that PASTE2 consistently achieved a higher average accuracy. Additionally, as the overlap percentage between adjacent slices in the dataset decreased, the accuracy of PASTE_alignment dropped rapidly, while PASTE2 consistently maintained a higher level of accuracy. This further emphasizes the ability of PASTE2 to align partially overlapped slices, which PASTE_alignment lacks.

To quantitatively compare the effectiveness of the four alignment methods, we introduced a coordinate-based accuracy score. This score was calculated by identifying MNN spot pairs based on the aligned coordinates of spots. We then determined the proportion of pairs where both spots belonged to the same type, serving as the accuracy metric. For the DLPFC, partial_DLPFC_0.85, and partial_DLPFC_0.7 datasets, we adjusted the number of neighbors considered during the MNN search to ensure that the proportion of spots found as MNN spots exceeded 80%, 70%, and 50%, respectively. Taking sample B as an example (Fig. 3c), we observed that in the DLPFC dataset, the alignment accuracy of PASTE_alignment, GPSA, and STAligner was essentially the same, with PASTE2 slightly outperforming them. However, as the percentage of overlap between adjacent slices decreased, the alignment accuracy for PASTE_alignment and GPSA deteriorated more rapidly. This finding highlighted the advantage of PASTE2 and STAligner in aligning partially overlapped slices, which further explained the alignment results for sample A in the DLPFC dataset. To provide a more intuitive demonstration of the alignment effects on partially overlapped datasets, we performed 3D visualization of the alignment results for sample C from the partial_DLPFC_0.85 dataset (Fig. 3d). This visualization confirmed that only PASTE2 and STAligner possessed the ability to align partially overlapped slices, thereby validating their effectiveness in such scenarios.

### 3.2 Results with data integration methods

**Fig. 4:**
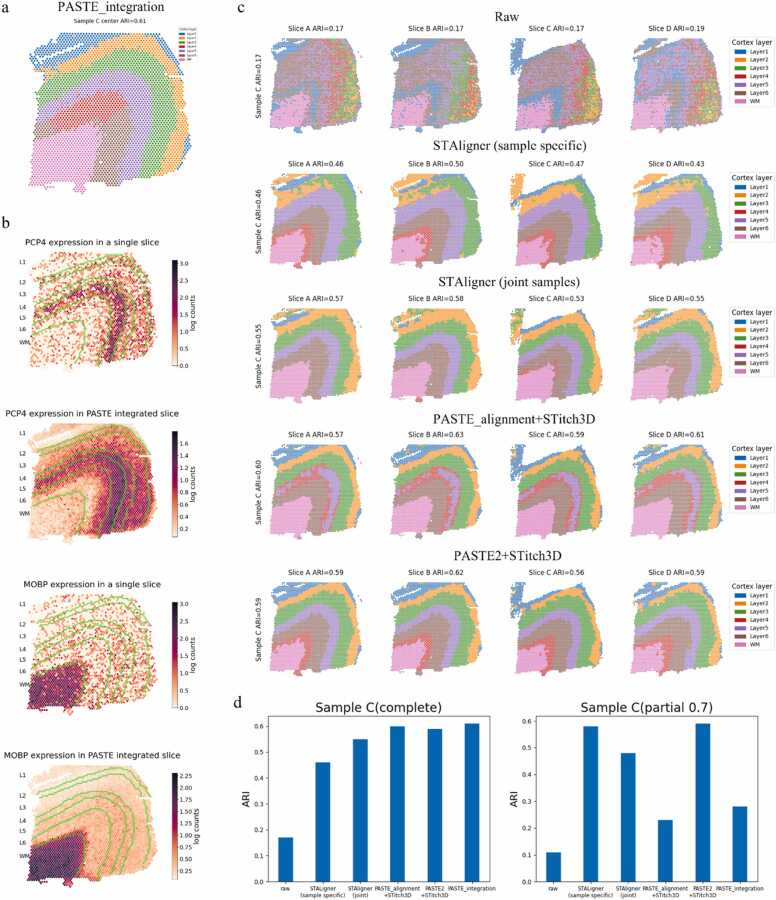
Experimental results with data integration methods tested on DLPFC dataset [56]**. a,** PASTE_integration applied on sample C from the DLPFC dataset using slice B as the template, generates a single center slice. The clustering result of the low-rank transcript count matrix using GMM is shown. **b,** The expression patterns of Pcp4 and MOBP in slice B of sample C and the center slice. The expression values vary more smoothly in the center slice. **c,** The clustering results for preprocessed slices of sample C from the dataset, without data integration, are displayed in the top row. The clustering outcomes of the data integration results using two modes of STAligner (sample specific & joint samples) and two modes of STitch3D (PASTE_alignment + STitch3D & PASTE2 + STitch3D) are shown either. **d,** The ARIs for the clustering results of sample C from the DLPFC and the partial_DLPFC_0.7 datasets, using the manual annotations of spots as ground truth.

We evaluated the performance of data integration methods, including PASTE_integration [44], STAligner [41], and STitch3D [43], on the DLPFC dataset (Fig. 4). To facilitate the analysis, we selected sample C as a representative example due to its unique structure, which made it a frequently used sample in our study. For STAligner, we examined two integration modes as outlined in the respective paper. The first mode, known as the sample-specific mode, involved integrating only the four slices of sample C. The second mode, called the joint-samples mode, entailed integrating all 12 slices from the three samples simultaneously. In the case of STitch3D, a pre-alignment step was necessary before performing data integration. To explore various integration approaches, we tested two methods for pre-alignment: PASTE_alignment and PASTE2.

Among the data integration methods evaluated, PASTE_integration emerged as a notable standout. This method effectively constructs a single center slice with the same shape as the pre-selected template by integrating a set of slices. Notably, this integrated center slice, which combines information from four independent slices, exhibited the remarkable effect of marker gene enhancement. By utilizing *Pcp4* and *MOBP* as marker genes, we observed their significant enrichment in layer 4–5 and the WM regions, respectively, within the center slice (Fig. 4b). Comparing the center slice with the slice B employed as the template, we noticed a smoother gene expression pattern in the integrated center slice. This enhanced coherence and smoothness make the center slice more suitable for gene expression enrichment analysis.

We applied Gaussian Mixture Model (GMM) [57] to cluster the integration results obtained from all the evaluated methods (Fig. 4a, c). For the raw data, clustering was performed using preprocessed gene expression matrices without any data integration. In the case of PASTE_integration, the low-rank transcript count matrix of the center slice was utilized as the low-dimensional representations of spots for clustering. For the other methods, we selected the low-dimensional representations of spots in the latent space for clustering. To quantitatively measure the effectiveness of data integration, we utilized manual annotations of spots as the ground truth and calculated the adjusted Rand index (ARI). Notably, all the evaluated methods exhibited significantly higher ARI compared to clustering using the original gene expression matrices. This indicates their capability to handle batch effects and preserve biological variations effectively. Among the evaluated methods, PASTE_integration demonstrated the highest ARI. However, it should be noted that PASTE_integration was unable to obtain a low-dimensional representation for each spot of the entire sample. In contrast, STitch3D exhibited similar performance, with both integration modes showing almost identical ARI. This similarity arises because, for datasets with high coverage between slices, PASTE_alignment and PASTE2 did not exhibit significant differences in alignment effectiveness. While STAligner demonstrated noticeable data integration compared to raw clustering, its performance was not as ideal, as evidenced by lower ARIs. The ARI achieved by integrating joint samples was superior to that obtained by integrating only sample C.

It is important to highlight that PASTE_integration did not consistently achieve the best results across all samples. In fact, in experiments involving sample A and sample B, PASTE_integration yielded the lowest ARI among all the data integration methods, indicating poor robustness when integrating all slices into a single center slice. However, STitch3D consistently demonstrated similar or significantly higher ARIs across all samples compared to STAligner. This can be attributed to STitch3D's more effective information communication across multiple slices, facilitated by the pre-alignment and 3D neighbor graph construction during the preprocessing stage.

To further compare the performance of these methods, we conducted similar tests on sample C from the partial_DLPFC_0.7 dataset and calculated the ARI (Fig. 4d). In contrast to the experiments conducted on the DLPFC dataset, both PASTE_integration and STitch3D with PASTE_alignment as the pre-alignment method exhibited a significant decrease in performance. This decline suggests that both PASTE_alignment and PASTE_integration struggle when dealing with partially overlapped datasets. However, STitch3D with PASTE2 as the pre-alignment method maintained a consistently high ARI in this context. This finding further emphasizes the critical role of constructing an accurate 3D neighbor graph for facilitating effective cross-slice information communication.

**Fig. 5:**
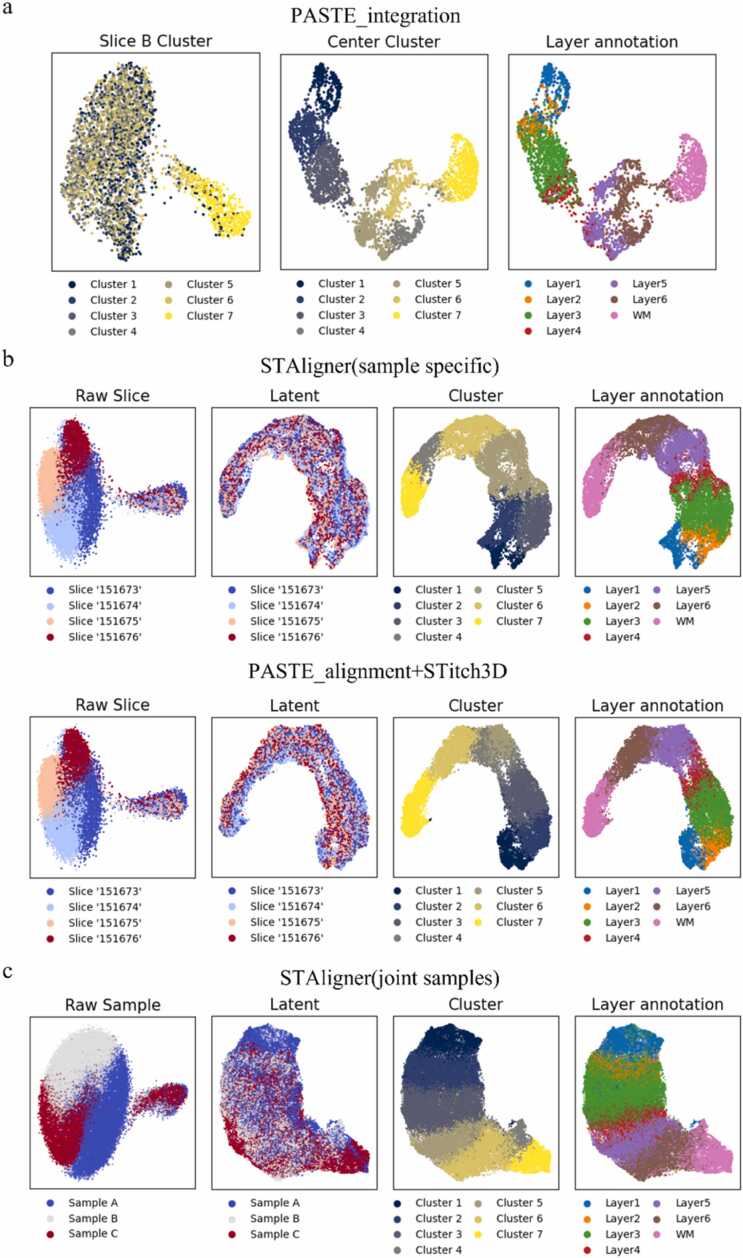
Visualization of the integration results on DLPFC dataset [56]**. a,** Visualization of the dimensionality reduction using UMAP for the raw data of slice B in sample C, which serves as the template (left), and for the low-rank transcript count matrix of the center slice obtained through PASTE_integration for sample C (middle and right). Spots are colored based on their respective clustering results and manual annotations. **b,** Integration results of four slices in sample C using STAligner and PASTE_alignment + STitch3D are visualized through dimensionality reduction. Different colors are assigned based on the slice indexes for the UMAP visualization of the raw data from the four slices. In contrast, colors are assigned based on slice indexes, clustering results, and manual annotations for the UMAP visualization of the representations in the latent space. **c,** Visualization of the dimensionality reduction using UMAP for the integration results of all the 12 slices in DLPFC dataset using STAligner. Different colors are assigned based on the sample indexes for the UMAP visualization of the raw data, while colors are assigned based on sample indexes, clustering results, and manual annotations for the UMAP visualization of the representations in the latent space.

We applied UMAP for dimensionality reduction and visualization of the results from data integration methods (Fig. 5). For PASTE_integration (Fig. 5a), we observed that prior to integration, it was challenging to identify spot types other than the WM since they were mixed together. However, after integration, spots from different types tended to gather further apart, allowing for improved recognition of additional spot types. Nonetheless, the identification of layer 2 and layer 4 spots remained challenging, as they were classified as other spot types. Through UMAP, we noticed evident batch effects between the four slices of the same sample (Fig. 5b). In contrast, both STAligner and STitch3D effectively mitigated batch effects in the data integration results for individual samples. This resulted in the mixing of spots from different slices, making them difficult to distinguish. However, STitch3D achieved better results compared to STAligner. It accurately identified different spot types, while STAligner misclassified layer 2 and layer 3 spots and erroneously separated the WM into two categories. Both methods encountered challenges in accurately identifying spots from layer 4. Similarly, noticeable batch effects were observed among the three samples (Fig. 5c). However, after performing joint integration using STAligner, spots from different samples mixed together more effectively. This indicated the successful removal of batch effects between different samples.

**Fig. 6:**
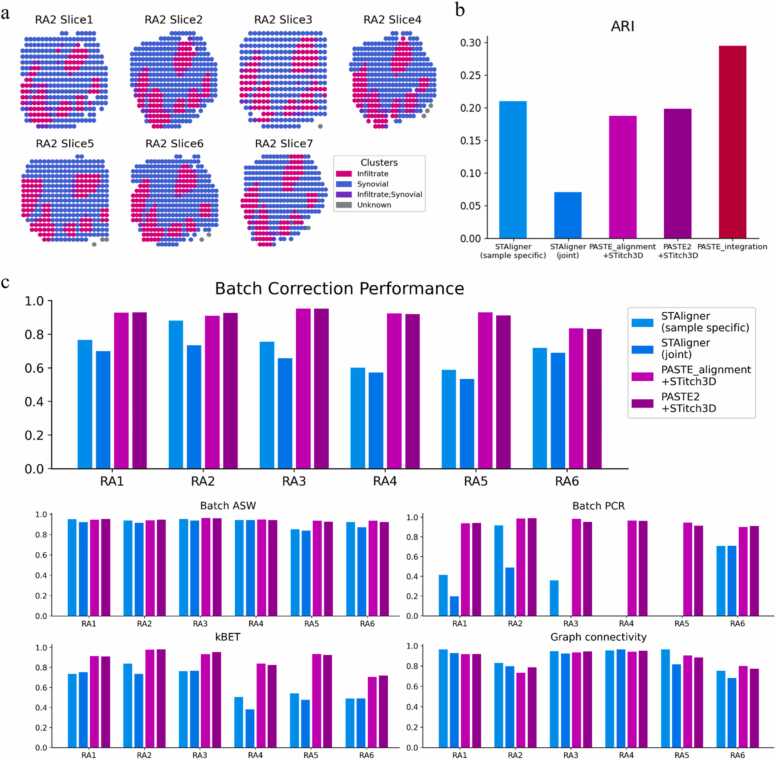
Experimental results with data integration methods tested on RA dataset [58]**. a,** Display of Sample RA2, the second sample among the six included in the RA dataset. Sample RA2 consists of seven slices, the highest among all six samples, and exhibits the most developed TLO-like structures. Spots are color-coded based on the annotations. **b,** Calculation of ARIs based on the clustering results after applying data integration procedures on RA2. The joint-samples mode of STAligner is exclusively used to integrate seropositive samples including RA1, RA2 and RA3. **c,** The quantitative comparison of the batch correction performance of the two modes of STAligner and two modes of STitch3D. Four metrics, including Batch ASW, Batch PCR, kBET, and Graph connectivity, are used to evaluate the effect of batch correction. The average score of these four metrics is used as the representation of batch correction efficacy (top). The joint-samples mode of STAligner is used twice, once to integrate all seropositive samples and once to integrate all seronegative samples.

In order to further explore the generalizability of different methods and mitigate the randomness of results caused by insufficient datasets. We further applied these data integration methods to a newly generated human rheumatoid arthritis (RA) synovium dataset [58], which is a more challenging scenario (Fig. 6). RA is an autoimmune disease with chronic inflammation in the synovium of the joint tissue [59], [60]. Within inflamed areas, there are localized accumulations of infiltrating leukocytes that organize into structures resembling secondary lymphoid organs (SLOs). These formations are commonly referred to as tertiary lymphoid organs (TLOs) histologically [61]. RA consists of two broad subtypes, seropositive and seronegative. These two types typically exhibit distinct biological differences, such as the presence of rheumatoid factor (RF) or anti-citrullinated protein antibodies (ACPA), which are only present in the seropositive subtype [58], [62].

The RA dataset consists of six samples from independent individuals, comprising three seropositive samples (RA1, RA2 and RA3) and three seronegative samples (RA4, RA5 and RA6). Each sample is accompanied by annotations of spots, which we used as the ground truth. Initially, we applied all the data integration methods mentioned above to integrate data from RA2 (Fig. 6a). RA2 consists of a total of seven slices, the highest among all six samples, and exhibits the most developed TLO-like structures [58]. For the joint-samples mode of STAligner, we only integrated the three seropositive samples and selected the corresponding representation for the spots from RA2. Subsequently, we clustered the spots based on the low-dimensional representation and calculated the ARI scores (Fig. 6b). On this sample, PASTE_integration achieved the best performance, while both modes of STitch3D and the sample-specific mode of STAligner yielded similar results. However, the joint-samples mode of STAligner yielded poorest result. We believe that the differences in performance can be attributed to the feature selection process used by each method. PASTE_integration utilizes genes that are common across all slices as selected features, resulting in a much larger number of selected genes compared to other methods. Therefore, in this experiment, it retains more biological information. Moreover, PASTE_integration can only obtain a single center slice, and clustering and ARI calculation are based solely on this template slice, making it more likely to achieve better results. STitch3D, on the other hand, selects features based on the chosen single-cell RNA reference, so the number and quality of selected genes largely depend on the quality of the selected single-cell RNA reference and its compatibility with the SRT dataset. In our experiment, we selected the single-cell RNA dataset [63] that was chosen as a reference by the original study of this dataset. STAligner, meanwhile, first selects highly variable genes (HVGs) for each individual slice and takes the intersection of these HVGs as the input for the model. This selection procedure is not ideal when there is significant noise between different slices or samples, as there may be too little overlap between different groups of HVGs. Consequently, this can result in insufficient retention of adequate biological information and lead to poor integration results. In our experiment, although we only integrated three seropositive samples when testing the joint-samples mode of STAligner, the selected HVGs were still only around 100, resulting in suboptimal integration results.

Furthermore, to quantitatively compare the batch correction capabilities of various data integration methods, we compared the integration results of all six samples in the RA dataset using four metrics provided by the *scib* package [64], including Batch ASW, Batch PCR, kBET, and Graph connectivity (Fig. 6c). The scores of these four metrics were averaged to represent the overall performance score of batch correction. We did not compute scores for PASTE_integration because it does not inherently possess batch correction capabilities, and it cannot provide a corresponding low-dimensional representation for each spot, making it impossible to calculate these scores. We found that all four modes of STAligner and STitch3D exhibited similar performance in terms of Batch ASW and Graph connectivity across each individual sample. However, the two modes of STitch3D outperformed the two modes of STAligner noticeably in terms of Batch PCR and kBET, resulting in a significantly higher overall score for STitch3D compared to STAligner for each sample. This can be attributed to the pre-alignment process of slices in STitch3D before integration and the establishment of the adjacency matrix in three-dimensional space, which strengthens the connections between different slices. This further corroborates the conclusion we drew earlier.

## 4 Conclusion and outlooks

We have reviewed the slice alignment and data integration methods for SRT data analysis. We have also compared them and offered our understanding of their performance. The comparison of capabilities of different methods is provided in Table 1.

**Table 1:** Comparison of the capabilities of different methods.

	PASTE	PASTE2	GPSA	STAligner	STitch3D
Slice alignment	√	√	√	√	×
Data integration	√	×	×	√	√
Align partially overlapped datasets	×	√	×	√	×
Non-rigid coordinate transformation	×	×	√	×	×
Provide representation for all spots	×	×	×	√	√
Integrate partially overlapped datasets	×	×	×	√	Need proper pre-alignment
Batch effect removal	×	×	×	√	√ (better)
Cell-type deconvolution	×	×	×	×	√

For slices alignment methods, PASTE_alignment demonstrates superior performance on datasets with highly overlapped slices, although it struggles with partially overlapped datasets. PASTE2 and STAligner are often more effective in aligning slices with varying coverage, despite some limitations in the estimation process of overlap percentage and the MNN-based alignment method. GPSA is the only method capable of flexible non-rigid coordinate transformations. However, it exhibits higher complexity, requires more time and memory, limiting its scalability.

Regarding data integration methods, PASTE_integration cannot provide low-dimensional representations for all spots and only generates a single center slice. This integration approach exhibits unsatisfactory robustness, and struggles to integrate datasets with low inter-slice coverage. STAligner and STitch3D, on the other hand, obtained low-dimensional representations for each spot in the latent space, attenuating batch effects and extracting biological information for downstream tasks. However, the pre-alignment step in STitch3D, contributing to improved cross-slice information communication, resulted in better handling of batch effects. Additionally, STitch3D has the capability for cell type decomposition, which STAligner lacks.

To further develop more efficient methods for establishing associations across multiple slices, several avenues can be explored. First, the utilization of subgraph-based approaches could be employed to enhance the efficiency of slice alignment and data integration. This would help reduce the time and memory consumption involved, ultimately improving scalability. Second, the introduction of adversarial training techniques could facilitate domain adaptation, mitigating the issue of over-alignment of slices. This approach would enable more effective handling of batch effects, leading to improved integration outcomes. Moreover, with the development and advancement of techniques aimed at preserving 3D positional anatomy at cellular resolution [58], [65], there arises a need for methods capable of directly processing data in a 3D view [66]. Such methods can leverage the true value of 3D SRT data, thereby forming a more comprehensive perspective. Lastly, as other branches of spatial omics technology continue to evolve, such as spatial epigenomics [67], there is an opportunity to extend the techniques of slice alignment and data integration to other spatial omics or multi-omics domains. Initial methods have already been developed in this direction [68], [69], and further exploration and refinement of these methods can help leverage the full potential of spatial omics technologies. By pursuing these avenues, we can expect to enhance the efficiency, effectiveness, and applicability of methods for integrating and analyzing spatial omics data across multiple slices.

## CRediT authorship contribution statement

**Yuyao Liu:** Writing – original draft, Writing - review & editing, Investigation, Visualization, Formal analysis, Data curation. **Can Yang:** Writing - review & editing, Methodology, Supervision.

## Declaration of Competing Interest

The authors declare that they have no conflicts of interest related to this research.
